# Polymerized ionic liquid Co-catalysts driving photocatalytic CO_2_ transformation†

**DOI:** 10.1039/d4su00194j

**Published:** 2024-07-17

**Authors:** Lisa Eisele, Bletë Hulaj, Maximilian Podsednik, Francesco Laudani, Pablo Ayala, Alexey Cherevan, Annette Foelske, Andreas Limbeck, Dominik Eder, Katharina Bica-Schröder

**Affiliations:** a Institute of Applied and Synthetic Chemistry, TU Wien Getreidemark 9/163 1060 Wien Austria katharina.schroeder@tuwien.ac.at; b KAI Kompetenzzentrum Automobil- und Industrieelektronik GmbH Argentinierstraße 8 1040 Wien Austria; c Analytical Instrumentation Center, TU Wien Lehargasse 6/Objekt 10 1060 Wien Austria; d Institute of Materials Chemistry, TU Wien Getreidemarkt 9/165 1060 Wien Austria; e Institute of Chemical Technologies and Analytics, TU Wien Getreidemarkt 9/164 1060 Wien Austria

## Abstract

Photocatalytic production of CO from CO_2_ has the potential for safe and atom-economic production of feedstock chemicals *via in situ* carbonylation chemistry. We developed novel ionic liquid-based polymeric materials through radical copolymerisation of 1-butyl-3-vinylimidazolium chloride and photocatalytically active Re- and Ru-complexes that serve as the CO_2_ reduction catalyst and photosensitiser, respectively. The crosslinked polymeric framework allows for the facile immobilisation of molecular organometallic complexes for use as heterogenised catalysts; moreover, the involved imidazolium core units co-catalyze the reduction of CO_2_*via* covalent interaction. The ratio of sensitiser and catalyst was analysed by laser ablation inductively coupled plasma mass spectroscopy (LA-ICP-MS) and set in relation to results from photocatalytic experiments. Ultimately, the heterogenous polymeric framework showed high selectivity for CO formation on photocatalytic CO_2_ reduction with improved stability to the corresponding homogenous system.

## Abstract 2

An ionic liquid polymeric matrix is used to immobilize a ruthenium photosensitizer and rhenium catalyst for selective CO_2_ reduction to CO.

## Graphical abstract

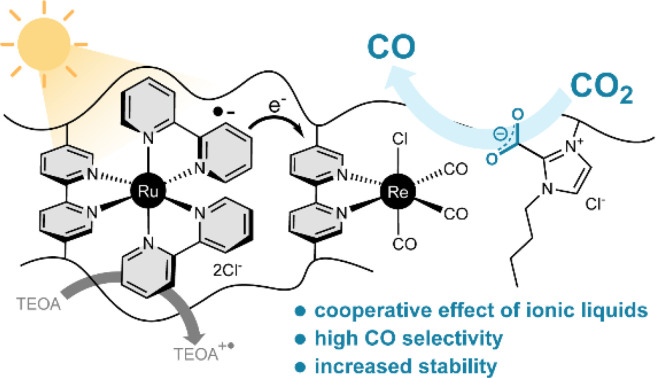

**Sustainability statement**

Facing the challenges of climate crisis, there is an urgent demand for technologies that drive the advancement of a circular economy using CO_2_ as a sustainable feedstock in chemical industries. Carbon dioxide can be reduced to CO, which serves as a feedstock for multiple processes. Combining photocatalyst with ionic liquids that can chemisorb and activate CO_2_ opens new pathways to efficient systems. In this context, we envisioned the development of a heterogenous, polymerized ionic liquid framework bearing photocatalytic groups for efficient and selective photocatalytic CO_2_ reduction. The aim of this work goes in line with the following UN Sustainable Development Goals: responsible consumption and production (SDG 12), climate action (SDG 13) and industry, innovation and infrastructure (SDG 9).

## Introduction

In light of the escalating levels of atmospheric carbon dioxide and the pressing challenges posed by the climate crisis, there is an urgent demand for technologies that can drive the advancement of a circular economy using CO_2_ as a sustainable feedstock.^1,2^ A crucial technology in this endeavour involves producing C1 building blocks from CO_2_, ultimately facilitating the formation of more intricate compounds.^3^ Carbon monoxide (CO) produced by electro^4^ or photochemical^5^ means emerges as an attractive feedstock for hydroformylation,^6^ Fischer–Tropsch synthesis^7^ and synthesis of carbonylated platform chemicals.^8^

An essential stage in CO_2_ reduction involves the challenging activation and first electron transfer, which requires a significant amount of activation energy and is accompanied by a change in the molecule's bond angle.^9^ Moreover, CO_2_ uptake is often limited by its solubility in the chosen reaction media.^10^ Ionic liquids (ILs) demonstrate an exceptional capability to absorb substantial amounts of CO_2_ through both physical (physisorption) and chemical (chemisorption) processes.^11,12^ Particularly noteworthy is the role of chemisorption in imidazolium-based ionic liquids as it also plays a crucial role in the activation of CO_2_.^13^ The formation of a carbene complex between ionic liquids and CO_2_ facilitates the bending of CO_2_. For imidazolium-based ionic liquids, the interaction occurs at the C2-position of the imidazole core, leading to the formation of an N-heterolytic carbene (NHC)–CO_2_ adduct.^14,15^ This interaction between ionic liquids and CO_2_ can efficiently reduce the overpotential of CO_2_ reduction, as confirmed by electrochemical experiments.^16,17^ To further reduce CO_2_ into valuable building blocks such as CO, both organo-metallic and purely inorganic photocatalytic catalytic systems have been proven to be powerful tools using solar light for the reduction of CO_2_.^5,18–21^

The combination of potent molecular systems with ionic liquid-based CO_2_ absorption and activation enables the photoreduction of CO_2_ with high selectivity under mild conditions, as our group and others have previously demonstrated for homogenous systems.^22–25^ Transferring such systems into heterogeneous materials is crucial facing factors like stability of metal–organic catalysts and catalyst handling on bigger scales following the goal of developing gas phase reactions as a big milestone in the development of CO_2_ capture and usage technologies.^26,27^

Out of a huge assortment of molecular systems consisting of metal–organic photosensitiser and catalysts systems ruthenium sensitizer and rhenium catalyst first introduced by Lehn have been established as a well-investigated benchmark system for CO_2_ reduction selective for CO reduction despite all efforts of replacing noble metal complexes by base metal ones such as iron, nickel and cobalt systems.^5,28,29^

Different approaches to incorporate the catalyst system with ruthenium sensitiser and rhenium catalyst in a heterogeneous matrix have been investigated, such as polymer frameworks and highly coordinated structures like COF and MOF-based systems.^30–32^ Direct polymerisation of vinyl-modified monomers is a straightforward method to incorporate ionic liquids into the polymer structure. Furthermore, special separation of transition metal reaction centres in heterogenous systems can be beneficial for long-term stability in comparison to homogenous systems, as dimer formation might be inhibited.^33^

Despite these obvious advantages, studies on immobilisation in a polymerizable ionic liquid matrix are rare. Zhang *et al.* studied materials based on styrene-linker functionalised catalyst with B12-catalyst and ruthenium tris-bipyridine sensitiser co-polymerized-IL for application in dechlorination reaction.^34^ The application of crosslinked bipyridine linkers was shown to allow for facile polymerisation. Zhou *et al.* tested a system based on the catalyst [Re(bpy)(CO)_3_]Cl with divinyl functionalisation of the bipyridine ligand and 1-ethyl-3-vinyl imidazolium bromide ionic liquid in CO_2_ reduction reaction and could prove high selectivity for CO. In this case, however, direct photoexcitation of the catalyst took place, leading to a limited light-harvesting capacity.^35^ In contrary to this photosystem, the introduction of a separate sensitiser moiety is beneficial as it can lead to absorption of photons at higher wavelengths, and additionally, prevent the damage of the catalyst during the redox cycling.

We previously showed that imidazolium-based ionic liquids exhibit a strong co-catalytic effect in the photocatalytic reduction of CO_2_ with an established photochemical system, consisting of a rhenium photocatalyst and a ruthenium photosensitiser. In this contribution, we integrate the advantageous attributes of imidazolium-based ionic liquids with this well-established photochemical system, consisting of a Re photocatalyst and a Ru photosensitiser, within an innovative crosslinked polymeric framework (CLP) to create an efficient method for selective visible-light CO_2_ photoreduction under benign reaction conditions.

## Results and discussion

The co-catalytic properties of the crosslinked polymer backbone are highly dependent on the design of the ionic liquid moiety. We selected ionic liquids with an imidazolium core structure since prior studies showed that such ionic liquids are capable of absorbing and activating high quantities of CO_2_*via* N-heterocyclic carbene (NHC) formation. A butyl side chain was introduced as a compromise to the long alkyl chains that offer increased CO_2_ uptake and short chain derivates with lower viscosity and reduced steric hindrance.^24,36^

The anion plays a key role in the deprotonation of the acidic proton on the C2-position of the imidazolium ring and the consecutive formation of the NHC–CO_2_ adduct. As previously shown, the co-catalytic effect of various imidazolium-based ionic liquids with different counter anions in CO_2_ reduction with ruthenium sensitiser and rhenium catalyst correlates with the Kamlet–Taft parameter β of the ionic liquids, thus highlighting the importance of hydrogen bond acceptor properties of the anion.^37^ Consequently, imidazolium-based ionic liquids with chloride as anion exhibited the best performance among all halogenides. Due to this superior performance and the presence of Cl as chlorido ligand in the ruthenium catalyst, we selected the ionic liquid 1-butyl-3-vinylimidazolium chloride (IL-1) as monomer for the design of the polymeric framework.^38^

**Fig. 1:**
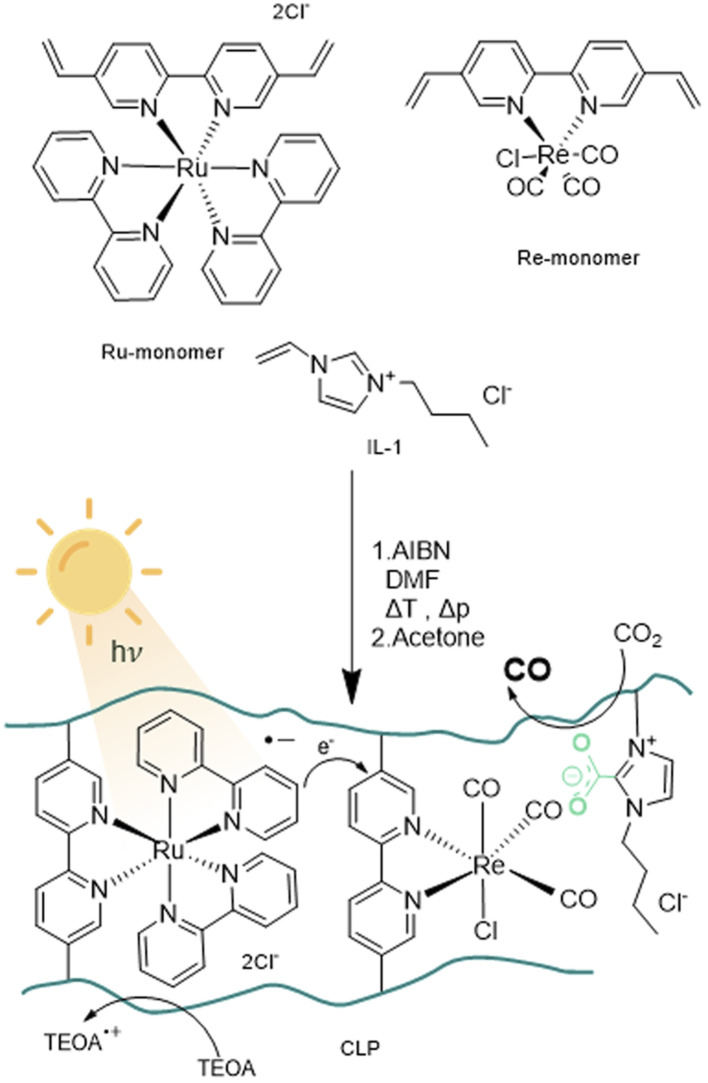
Schematic representation of monomers used for the radical polymerisation of crosslinked polymers (CLP) with AIBN (azobisisobutyronitrile) towards a polymer framework for photocatalytic CO_2_ reduction.

The design of the catalyst relies on a cross-linked polymeric framework as illustrated in Fig. 1, where Ru centres can act as visible-light-absorbing moieties and transfer excited electrons to the Re centres. For this purpose, the bipyridine ligands of the Re-containing photocatalyst monomer and the Ru-containing photosensitizer monomer, were modified with vinyl substituents *via* palladium catalysed Suzuki–Miyaru coupling mechanism.^39^ The modified ligand was than introduced to the complex by ligand exchange from *cis*-bis(2,2′-bipyridine) dichloro ruthenium(ii) and pentacarbonylchloro rhenium(i).^35^

With the desired monomers in hand, polymerisation was performed *via* free radical polymerisation with azobisisobutyronitrile (AIBN) as a radical starter under pressurized conditions. The ratio of monomers was chosen in variable ratios of catalyst and sensitiser with excess of ionic liquid, resulting in solid, hygroscopic crosslinked polymeric frameworks (CLP) that were isolated after precipitation with acetone (Table 1, Batch CLP-1 to CLP-4).

**Table 1:** Theoretical and experimental values for the composition of cross-linked polymers CLP-1 to CLP-4 with variable content of Ru and Re

Entry		Ratio		wt% Re		wt% Ru	
Batch	Theo.^a^	ICP-MS^b^	T-XRF^c^	Theo.	ICP-MS^b^	Theo.	ICP-MS^b^
CLP-1	1 : 10 : 90	19.31	14.23	0.77	0.58	4.17	6.03
CLP-2	1 : 0 : 90	0.00	0.00	1.08	0.34	0.00	0.00
CLP-3	1 : 10 : 90	8.10	7.61	0.77	0.55	4.17	2.42
CLP-4^d^	1 : 10 : 90	10.26	12.36	0.77	0.65	4.17	3.63

a Molar ratio of monomers Re-monomer : Ru-monomer : IL-1.

b Measured by laser ablation inductively coupled plasma mass spectroscopy (LA-ICP-MS). Calculated from average of 6 lines scanned 3 times. Quantification with external standard set into relation to quantification of microwave digested aliquots of CLP-3. RSD ≤ 5%.

c Calculated from total reflection X-ray fluorescence spectroscopy (T-XRF) measurements.

d Upscaled preparation by factor 5.

### Material characterisation

Successful incorporation of Re-monomer and IL-1 was initially proven by Fourier transform infrared spectroscopy (FT-IR) *via* comparison of the spectral data recorded from IL-1, polymerized ionic liquid (PIL), Re-monomer and the polymer CLP-1 (Fig. S1†). Signals between 2025 and 1870 cm^−1^ (shaded area) correspond to the stretching vibrations of CO ligands of the Re complex and are visible in the spectra of the Re-monomer and the CLP. The vibration of the imidazole ring of the ionic liquid occurs at 1465 cm^−1^, while the CH vibrations of the imidazole ring correspond to the signal peaks at 1542 and 1567 cm^−1^. Therefore, it can be safely concluded that the Re-photocatalyst and ionic liquids are appropriately built into the polymer frameworks.

Thermogravimetric analysis (TGA) was performed on the polymerised ionic liquid (PIL) without Ru and Re, as well as polymer containing Re-monomer (CLP-3) and mixed polymer CLP-4 (Fig. S2†). All samples show an initial small mass loss starting at 100 °C, referring to the loss of absorbed water. From 240 °C, organic matter starts to decompose, leading to significant relative mass loss for all three samples. This loss is accordingly reduced in ruthenium or rhenium due to their higher inorganic content.

**Fig. 2:**
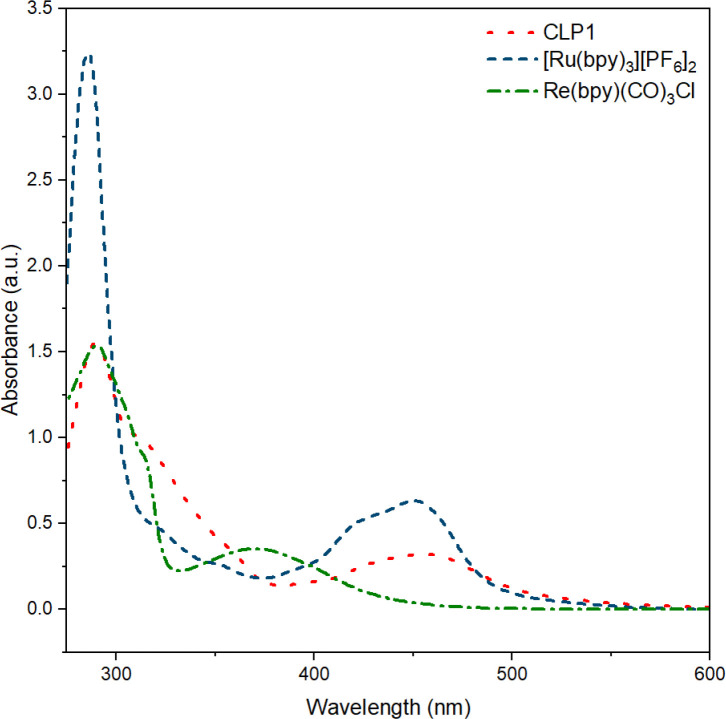
UV-vis spectra of polymer CLP-1 (red, dot), and complexes [Ru(bpy)_3_][PF_6_]_2_ (blue, dash) and Re(bpy)(CO)_3_Cl in MeCN (green, dash-dot).

UV-vis measurement on CLP-1 and monomer samples further proves the successful incorporation of photo redox-active monomers Ru-monomer and Re-monomer (Fig. 2). The broad signal between 400 and 500 nm can be attributed to the metal-to-ligand charge transfer (MLCT) transition of Ru-monomer.^40^ The maximum of this transition is red-shifted by around 5 nm. The shoulder at around 350–400 nm can be attributed to Re MLCT, that is blue shifted compared to the free complex. Both red shift of Ru and blueshift of Re might be an indicator for spectral interaction between the two species within the polymer framework.

**Fig. 3:**
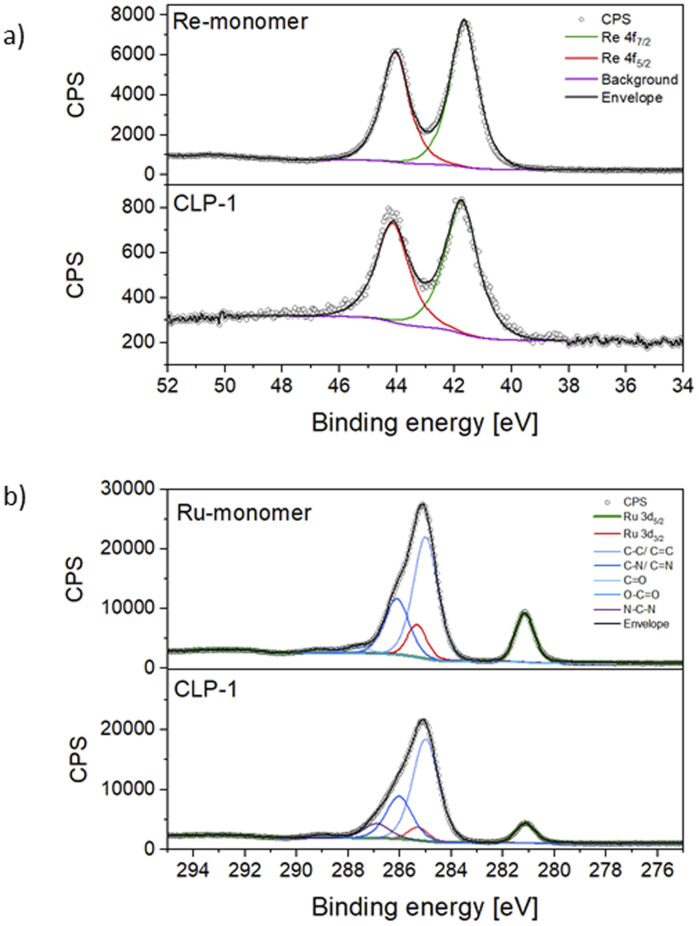
Detailed XPS scans of CLP-1 and monomers. Re 4f (a) and C 1s together with Ru 3d_5/2_ (b).

For a deeper understanding of the oxidation states of Ru photosensitizer and Re-catalyst in the samples, X-ray photoelectron spectroscopy (XPS) on CLP-1 and both Re-monomer and Ru-monomer was performed. The survey spectrum of CLP-1 clearly detects O 1s, C 1s, N 1s, Cl 2p and Re 4f along with Ru 3d (Fig. S3†). Comprehensive scans of Re 4f in Re-monomer reveal signals corresponding to Re 4f_7/2_ and 4f_5/2_ at 44.0 and 41.60 eV respectively, consistent with data reported for Re(i) oxidation state (Fig. 3a).^41^ Comparing Re-monomer with CLP-1 the Re 4f signal is not shifted (<0.1 eV) (Fig. 3a). This evidence confirms the integrity of the catalytic site throughout the polymerization process. The clear separation of Ru 3d_5/2_ from C 1s is beneficial for the detection of Ru in the polymer framework. The peak maximum for Ru 3d_5/2_ can be detected at 281.1 eV for the Ru-monomer and CLP-1, which is in accordance with data from the literature for Ru(ii).^42^

Scans of the Cl 2p peak show different positions for the two monomers due to the different bonding state (Fig. S4†). The Cl 2p scans for the Ru monomer show two different chemical states, which have been assigned as anionic and covalently bonded. The anionic component is quantitatively dominant and the peaks' positions perfectly match with the ones measured for the CLP-1 polymer (196.7 eV and 198.3 eV).^43,44^ The Cl 2p peaks from the Re catalyst are shifted 1.0 eV higher due to their ligands bonding nature but they don't appear in the polymer scans. This is most likely due to a negligible contribution coming from the much lower concentration of Re detected in XPS respect to the Ru one. Comparing the N 1s spectra of CLP-1 with those of both Re-monomer and Ru-monomer facilitates the distinct identification of nitrogen peaks originating from imidazole and bipyridines in the N 1s spectrum of CLP-1 (Fig. S5†). Bipyridine peaks can be identified at 400.1 eV (Re-monomer), 400.2 eV (Ru-monomer) and 400.1 eV (CLP-1) whereas the imidazole peak is shifted to higher binding energies (401.5 eV) in CLP-1.^45–47^ The deviation between N 1s bipyridine peaks in CLP-1 and Ru-monomer can be explained by the measurement error in energy of 0.1.

**Fig. 4:**
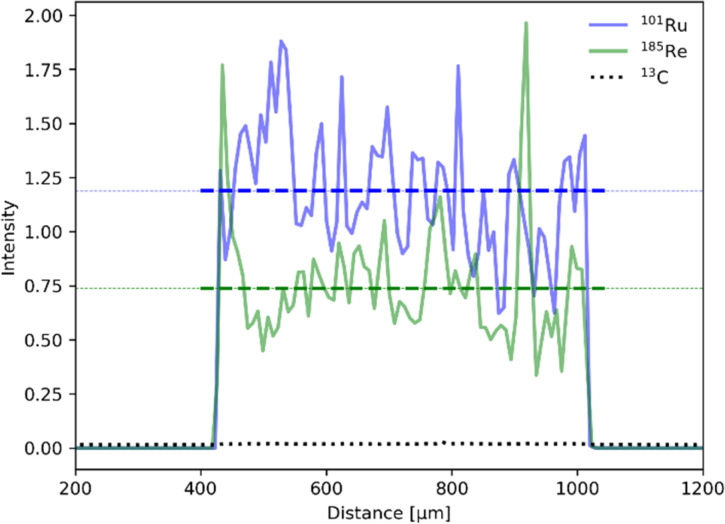
LA-ICP-MS line scan for ^101^Ru (red) and ^185^Re (green) normalized to the ^13^C isotope (black, dotted). Average intensity for each isotope is depicted as dotted line.

Establishing the absolute Ru and Re contents in the polymer is crucial for the evaluation and benchmarking of catalytic properties of our CLP and was therefore further investigated by laser ablation inductively coupled plasma mass spectroscopy (LA-ICP-MS). The profile of the line scans reveals an even distribution of Ru and Re within the sample particles (Fig. 4). Scans of all elements are shown in the ESI (Fig. S6†). The uniformity of the particle morphology was further confirmed by scanning electron microscopy (SEM) (Fig. S7†), thus overall suggesting that the elements Ru and Re are evenly incorporated during the polymerization process. Exact quantification of Ru and Re in the samples was performed after standardisation with a sample completely dissolved by microwave digestion under acidic conditions. It is worth noticing that the Ru content varied in the range of 2–6 wt% for 3 polymer batches, whereas results for Re were far more consistent and averaged around 0.6 wt%. The ratio of Ru and Re was further confirmed by T-XRF (total reflection X-ray fluorescence spectroscopy) measurements (Table 1). The variability can be attributed to the inherent unpredictability of free radical polymerization, leading to inconsistent insertion of the monomers.

### Photocatalytic CO_2_ reduction

Photocatalytic testing was performed in a quartz glass reactor using organic solvents bubbled with CO_2_ and the sacrificial agent triethanolamine (TEOA). The yield of CO, H_2_ and CH_4_ was traced by headspace gas chromatography under illumination with *λ* = 445 nm. Testing of CLP-1 in 15 h-long experiments revealed photocatalytic activity up to turnover numbers (TON) of 60, producing 0.7 μmol of CO in acetonitrile (MeCN) as solvent (Table 2). Comparing the performance of CLP-1 in dimethylformamide (DMF) showed that the reaction rate is significantly lower than in MeCN. A possible explanation for this observation could be the swelling behaviour of the polymer, affecting the accessibility of reaction centres.

**Table 2:** Photocatalytic CO_2_ reduction with cross-linked polymers

Entry	Batch	Cond.^a^	Ru [wt%]	CO^b^ [μmol]	TON^c^	Sel. CO^d^ [%]
1	CLP-1	445 nm, MeCN	6.03	0.70	60	97.7
2	CLP-1	445 nm, DMF	6.03	0.08	7	97.6
3	CLP-1	500 nm, MeCN^e^	6.03	0.71	61	97.8
4	CLP-1	445 nm, MeCN^f^	6.03	0.03	3	81.2
5	CLP-2	445 nm, MeCN	0.00	0.17	1	98.3
6	CLP-2	365 nm, MeCN	0.00	0.01	0	n.d.
7	CLP-3	445 nm, MeCN	2.42	0.33	28	>99

a Conditions: 0.3 mg mL^−1^ polymer, 0.32 M TEOA, *T* = 22 °C, *t* = 15 h, standard solvent MeCN, 5% brightness.

b Yields calculated from headspace GC sampling.

c Turnover number (TON) calculated for.

d Selectivity calculated for CO.

e Bandpass filter 500 nm adjusted photon flux.

f Brightness mode 100% (20-fold photon flux of condition a).

A set of experiments with different illumination settings was further carried out to study the excitation mechanism of the system. Illumination at 445 nm leads to a TON of 60 and yields 0.7 μmol of CO. Repetition of the experiment under broad visible spectrum (see experimental part) using a 500 nm bandpass filter (emission range: 472–527 nm) yielded similar values (0.7 μmol and TON = 61), which further indicates that major excitation takes place *via* Ru centres. This is in line with the proposed excitation and charge transfer mechanisms (Fig. 1), where Ru is excited and quenched with TEOA to be the one-electron-reduced species. Electrons are then transferred from Ru-centres to Re-centres *via* orbital overlap through the partially conjugated backbone or trough collision of two units in close proximity in order to start the CO_2_ reduction reaction on the Re-reaction centre. Reaction mechanisms relying on Re-excited states may play a less relevant role, as direct excitation of Re seems to be less efficient under the chosen conditions. Alternatively, Re-excited states might form upon energy transfer from Ru-excited states.

Given the partial conjugation of the polymer backbone, we anticipate the potential for energy transfer through Fluorescence Resonance Energy Transfer (FRET) or Dexter transfer mechanisms for exchanges between Ru-monomer and Re-monomer. FRET involves a dipolar mechanism, where two dipoles are coupling non-radiatively, while Dexter energy transfer relies on electron transfer through orbital overlap *via* a conjugated system (intramolecular) or physical contact of reaction partners (intermolecular). FRET and Dexter mechanism differ in the length scale of the effects to occur. FRET can take place over a distance of 100 Å whereas intramolecular Dexter transfer is limited to a radius of 10 Å. Besides energy transfer the transfer of electrons can occur as well *via* a Dexter like mechanism over longer distances compared to energy transfer.^48^ The ratio and quantity of ionic liquid will determine the average distance between the Re-monomer and Ru-monomer units. Based on the selected ratio, it is reasonable to conclude that the average distance between Re-monomer and Ru-monomer units within the framework exceeds the 10 Å radius required for intramolecular Dexter energy transfer to take place. Therefore, it can be assumed that FRET, as a mechanism operational over 10–100 Å, is the dominating mechanism for the transfer of excited electrons, as well as Dexter-like electron transfer *via* orbital overlap. A substantial decrease in the product yield with increased photon flux (20-fold increase) indicates the fragility of the sensitizer towards light-induced ageing and suggests that a balance between photoexcitation and redox charge transfer needs to be maintained to avoid degradation (Table 2). The effect of photosensitizer ageing seems to outperform possible kinetic limitations of CO_2_/CO absorption and desorption as the rate-limiting step as the reaction rate of entry 1 couldn't be reached.

Additional experiments in the absence of Ru-monomer with CLP-2, at excitation wavelengths 365 nm and 445 nm led only to traces of CO and H_2,_ proving the crucial role of Ru monomer as a sensitizer. The impact of ruthenium content on the mechanism was investigated by comparing the performance of CLP-1 and CLP-3 samples, where CLP-3 has half the amount of Ru-monomer as measured by LA-ICP-MS. The increase in Ru-monomer from 2.42 wt% to 6.03 wt% led to the rise of TON per Re centre from 28 to 60, which strongly suggests that another balance between the number of Ru and Re centres needs to be established to achieve optimal charge utilization. A higher content of Ru-monomer within the polymer framework might lead to a shorter average distance between Ru and Re centres and a higher availability of electrons for photoreaction on the Re centres. Both samples exhibit excellent selectivity for CO.

**Fig. 5:**
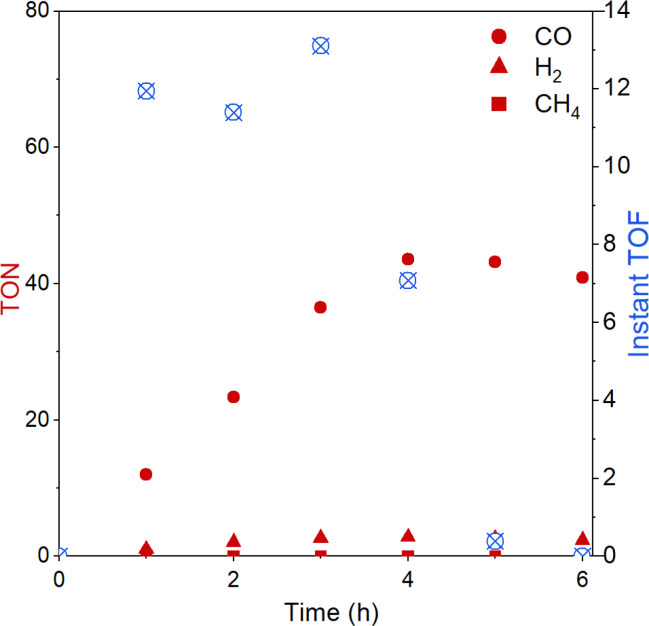
Turnover number (TON) and instant turnover frequency (instant TOF) of CO_2_ reduction reaction over the time course of 6 h with CLP-1.

The evolution of CO over time was further studied with the best-performing polymer CLP-1 over 6 hours (Fig. 5). Evaluation of instant TON and TOF reveal that the system is stable over 4 hours. It is interesting to compare this to results from homogenous photocatalysis that we carefully studied in our previous work.^25^ There we also observed stable performance followed by a sudden deactivation, however this took place already after 40 min under the conditions otherwise similar (light, intensity, solvent). A possible explanation for the decrease in catalytic activity is the light-induced degradation of photosensitiser.^49^ This fragility can be further confirmed by UV-vis spectroscopy of the reaction solution after illumination (Fig. S8†). A strong drop in absorption in the range from 440 to 445 nm accompanied by a red shift of the signal assigned to the MLCT indicates the degradation of Ru sensitizer. Additionally, the degrease in absorption intensity below 380 nm indicates additional degradation of Re catalyst. Despite CLP-1 is ultimately deactivated, we see a positive effect of the catalyst heterogenization which is able to prolong the stable performance 5 times resulting in instant turnover frequencies up to 13 min^−1^.

## Conclusion

In this study, we present the successful synthesis of heterogeneous polymerized ionic liquid frameworks incorporating immobilized ruthenium sensitizers and rhenium catalyst moieties for the selective photocatalytic reduction of CO_2_ to CO *via* radical polymerization with AIBN. The successful integration of ruthenium and rhenium moieties was confirmed through UV-vis spectroscopy, IR, and XPS analyses. Quantification of rhenium and ruthenium content was carried out using LA-ICP-MS, enabling facile determination of metal contents even on single crumbs of the sample. The immobilized rhenium content ranged from 0.34 to 0.65 wt%, with overall rhenium contents reaching up to 6 wt%. The synthesized samples were evaluated for their photocatalytic CO_2_ reduction activity, revealing high selectivity towards CO with turnover numbers (TON) reaching up to 60 and stable turnover frequencies (TOF) sustained over 4 hours. Notably, the high selectivity persisted throughout the entire 4 hours duration. The introduction of photosensitizer led to a significant enhancement in CO_2_ TON compared to samples lacking rhenium, with CO TON showing a linear increase corresponding to the amount of sensitizer incorporated into the polymer. Our future endeavours aim to develop systems with enhanced long-term stability, focusing on strategies applicable in the gas phase. Ongoing work in our laboratory is directed towards this objective.

## Materials and methods

All chemicals purchased from commercial suppliers were used without further purification unless otherwise noted. Dry solvents intended for anhydrous reactions were pre-distilled and desiccated on Al_2_O_3_ columns (PURESOLV, Innovative Technology). Column chromatography was carried out on standard manual glass columns using silica gel from Merck (40–63 μm) with pre-distilled solvents. TLC analysis was performed on precoated aluminium-backed plates from Merck (silica gel 60 F254). UV active compounds were visualised at 254 nm.

Infrared (IR) spectra were recorded on a PerkinElmer Spectrum 65 FT IR spectrometer equipped with a particular MK II Golden Gate Single Reflection ATR unit. UV-vis spectroscopy was performed on a JASCO V-670 spectrophotometer with a spectral test range of 190 to 900 nm. Thermogravimetric analysis was conducted on a Netzsch STA 449 F1 system. The temperature was increased from 25 °C to 500 °C with a rate of 5 °C min^−1^.

^1^H and ^13^C, NMR spectra were recorded on a Bruker Advance UltraShield 400 MHz spectrometer, and chemical shifts were reported in ppm using TMS (tetramethylsilane) as the internal standard. Coupling constants (*J*) are given in Hz. For NMR purposes, the multiplicities are reported using the following abbreviations: s = singlet, d = doublet, t = triplet, q = quartet, m = multiplet, dd = doublet of doublets, brs = broad singlet.

Headspace GC analysis was performed with the SHIMADZU Nexis™ GC-2030 gas chromatograph equipped with a dielectric barrier discharge ionisation detector (BID) and 2 m ShinCarbon ST column (Restek Co.)

Total-reflection X-ray fluorescence spectroscopy was performed using an Atomika 8030C X-ray fluorescence Analyser, which operates with a total reflection geometry using an energy-dispersive Si(Li)-detector. The measurements were performed with the tungsten continuous spectrum excitation mode (35 keV) at 50 kV and 47 mA for 100 s live time.

XPS Spectra were collected using a Physical Electronics PHI Versaprobe III with a hemispherical energy analyzer and a monochromatic aluminium Kα X-ray source (1486.6 eV).

In order to avoid contact with moisture and air sample transfer from the glove box into the vacuum of the XPS instrument was performed under argon atmosphere using a transfer vessel provided by PHI. Samples were attached to the sample stage with insulating double sided scotch tape. Charging was prevented through the instrument's charge neutralization system. Data were collected using a 200 μm, 50 W focused X-ray beam at a base pressure of 1 × 10^−9^ mbar, and a take-off angle of 45°. Survey scans were collected with a pass energy of 140.00 eV and a step size of 0.5 eV. High-resolution scans of peaks of interest were collected with a pass energy of 27.00 eV and a step size of 0.05 eV. Data were analyzed with CASA XPS software. All peaks were referenced to the C 1s aromatic component (285.0 eV). Peak fittings were performed with a Shirley-type background. Gaussian Lorentzian product (GL) and Lorentzian Asymmetric (LA) lineshapes have been used for the peak fittings: GL(50) (C 1s), LA(1.2,0.9,600) (Ru 3d), GL(80) (Re 4f), LA(80) (N 1s), LA(0.95,2,300) (Cl 2p). The pure monomers' fitted components have been used as a reference to constrain the same peaks in the polymer.

LA-ICP-MS measurements were performed using the “imageGEO193”, an ArF laser ablation system equipped with a “TwoVol3” ablation chamber from Elemental Scientific Lasers (Bozeman, MT, USA), coupled *via* a PTFE tubing to the “iCAP-Q” from ThermoFisher Scientific (Marietta, OH, USA). An 800 mL min^−1^ helium carrier flow was employed for the analysis, and samples were fixated on a microscope slide using adhesive tape. The samples were ablated using line scans across the polymer crumbs with the following laser parameters: 0.5 J cm^−2^ laser fluence, 20 μm spot size, 50 Hz frequency, and 100 μm s^−1^ scan speed. The ICP-MS was operated in standard mode, recording the following isotopes: ^13^C, ^100^Ru, ^101^Ru, ^102^Ru, ^104^Ru, ^185^Re, and ^187^Re. For the measured isotopes, a dwell time of 10 ms was used, and all the results were normalized to ^13^C. Before all experiments, the instrument was tuned to maximum intensity using SRM NIST126. An in-house prepared matrix-matched standard was used to quantify the Ru and Re content.

### Synthesis of monomers

1-Butyl-3-vinyl imidazolium chloride (IL-1) was synthesized according to literature.^38^

#### 5,5′-Divinyl-2,2′-bipyridine

5,5′-Dibromo-2,2′-bipyridine (4, 250.0 mg, 0.796 mmol, 1.0 equiv.), potassium vinyltrifluoroborate (397.8 mg, 2.95 mmol, 3.7 equiv.), palladium(ii) acetate (3.6 mg, 0.016 mmol, 0.02 equiv.), triphenylphosphine (11.1 mg, 0.042 mmol, 0.05 equiv.) and caesium carbonate (726.4 mg, 2.23 mmol, 2.8 equiv.) were sealed in a 20 mL microwave vial equipped with a stirring bar and placed under Ar atmosphere. Degassed THF (13 mL) and water (0.53 mL) were added *via* syringe. The reaction mixture was stirred under reflux for 24 h. After cooling to room temperature, water (14 mL) was added, and the mixture was extracted with EtOAc. The combined organic layers were dried over anhydrous sodium sulfate, after which the solvent was removed under reduced pressure. The resulting crude was charged on silica and purified *via* column chromatography (4 : 1 : 0.25 = PE : EtOAc : TEA, *R*_f_ = 0.4). The desired product was obtained as a colourless powder (116.6 mg, 70%).

^1^H NMR (400 MHz, CDCl_3_): *δ* 8.67 (s, 2H, H-arom), 8.37 (d, *J* = 8.3 Hz, 2H, H-arom), 7.86 (dd, *J* = 8.3, 2.3 Hz, 2H, H-arom), 6.77 (dd, *J* = 17.1, 11.0 Hz, 2H, –CH=CH_2_), 5.90 (d, *J* = 17.7 Hz, 2H, =CH_2_), 5.42 (d, *J* = 11.0 Hz, 2H, =CH_2_). ^13^C NMR (400 MHz, CDCl_3_): *δ* 155.19 (C-arom), 147.95 (C-arom), 133.60 (-CH=CH_2_), 133.46 (C-arom), 133.11 (C-arom), 120.92 (C-arom), 116.47 (=CH_2_). FT-IR (ATR, *ν* cm^−1^): 3000, 1627, 1589, 1465, 1363, 1024, 910 (C=C), 843.

#### Re-monomer

5,5′-Divinyl-2,2′-bipyridine (41.0 mg, 0.197 mmol, 1.0 equiv.), pentacarbonylchlororhenium(i) (71.3 mg, 0.197 mmol, 1.0 equiv.) and toluene (20 mL) were mixed under inert conditions in a single-neck flask equipped with a stirring bar. The reaction mixture was stirred under reflux for 16 hours. After cooling to room temperature, the solid was filtered off and washed with dry toluene.

The desired product was obtained as a yellow powder (81.3 mg, 80%) ^1^H NMR (400 MHz, DMSO): *δ* 8.98 (s, 2H, H-arom), 8.75 (d, *J* = 8.6 Hz, 2H, H-arom), 8.55 (dd, *J* = 8.6, 2.1 Hz, 2H, H-arom), 7.03 (dd, *J* = 17.8, 11.2 Hz, 2H, –CH=CH_2_), 6.29 (d, *J* = 17.7 Hz, 2H, =CH_2_), 5.68 (d, *J* = 11.2 Hz, 2H, =CH_2_). ^13^C NMR (400 MHz, DMSO) *δ* 153.97, 151.09, 136.35, 135.74, 131.24, 124.30, 120.91. FT-IR (ATR, *ν* cm^−1^): 2017 (CO), 1875 (CO), 1478, 1377, 1251, 915 (C=C), 856.

#### Ru-monomer

5,5′-Divinyl-2,2′-bipyridine (35.4 mg, 0.170 mmol, 1.0 equiv.), *cis*-bis(2,2′-bipyridine) dichloro ruthenium(ii) hydrate (9, 123.5 mg, 0.255 mmol, 1.5 equiv.) were sealed in a 20 mL microwave vial equipped with a stirring bar and placed under argon atmosphere. Dry ethanol (8.2 mL) and water (0.82 mL) were degassed and added *via* syringe. The reaction mixture was stirred under reflux for 16 h. After cooling to room temperature, the solvent was removed under reduced pressure. The resulting crude was dissolved in acetonitrile (5 mL) and precipitated in diethyl ether (100 mL). The solid was separated and dissolved in double-distilled water (5 mL). Insoluble residues were separated through a sintered glass filter. Acetonitrile (5 mL) was added to the filtrate, and then the solvent was removed under reduced pressure. The solid was dried over phosphorus pentoxide for 48 h to afford the desired product as a dark violet solid (115.1 mg, 98%).

^1^H NMR (400 MHz, MeOD): *δ* 8.73 (d, *J* = 9.3 Hz, 4H, H-arom), 8.65 (d, *J* = 8.5 Hz, 2H, H-arom), 8.27 (dd, *J* = 8.6, 2.0 Hz, 2H, H-arom), 8.18–8.12 (m, 4H, H-arom), 7.91–7.80 (m, 4H, H-arom), 7.68 (d, *J* = 2.0 Hz, 2H, H-arom), 7.56–7.48 (m, 4H, H-arom), 6.59 (dd, *J* = 17.7, 11.1 Hz, 2H, –CH=CH_2_), 5.88 (d, *J* = 17.6 Hz, 2H, =CH_2_), 5.48 (d, *J* = 11.1 Hz, 2H, =CH_2_) ^13^C NMR (151 MHz, MeOD) *δ* 158.46, 157.12, 152.74, 150.38, 139.31, 138.51, 135.24, 132.45, 129.00, 125.70, 120.91.

### General procedure for polymerisation

The required amounts of monomers, AIBN and DMF were sealed in an 8 mL screw-cap vial equipped with a stirring bar in the glovebox. The reaction was carried out in the autoclave purged with N_2_ for 24 h, at 100 °C, at elevated pressures. After cooling to room temperature, the product was precipitated in anhydrous acetone and dried *in vacuo* yielding red solids.

### Photocatalytic experiments

Photocatalysis was performed using a glass reactor built in house with a total volume of 3.7 mL, equipped with a septum, water-cooling system, and a stirring bar. A Solis High Power LED emitting light at 445 nm, powered by a DC2200 – High-Power 1-Channel LED Driver and set to a brightness of 5%, was employed as the light source. For experiments with 500 nm bandpass filter a Thor labs Solis 3c lamp was used in combination with a FBH500-40 hard-coated bandpass filter.

For photocatalytic experiments, polymer and TEOA were weighed into a Schlenk tube. The degassed and water-free solvent was added, and the sample was sonicated for three hours to dissolve the polymer completely. The solution was degassed *via* freeze–pump–thaw technique and transferred to the reactor. Before the reaction, reaction mixtures underwent CO_2_ purging at a rate of 10 mL min^−1^ for 3 minutes. Reaction products were traced by Headspace GC analysis. All samples were checked for sufficient CO_2_ saturation by GC detection of CO_2_ at *t* = 0 h and *t* = 15 h. Detailed descriptions of calculations of reaction yield, TON and Instant TOF are provided in S9.†

## Data availability

The data supporting this article have been included as part of the ESI.†

## Conflicts of interest

There are no conflicts to declare.

## Supplementary Material

SU-002-D4SU00194J-s001
