# Supplementary material for: Polymerized ionic liquid Co-catalysts driving photocatalytic CO2 transformation
Source: RSC Sustain. 2024 Jul 17;2(9):2524–31. doi: 10.1039/d4su00194j (PMC11353680; doi:10.1039/d4su00194j)
Supplement: SU-002-D4SU00194J-s001 [file SU-002-D4SU00194J-s001.pdf]

Supplementary Information (SI) for RSC Sustainability.
This journal is © The Royal Society of Chemistry 2024

Polymerized Ionic Liquid Co-Catalysts Driving Photocatalytic CO_2 Transformation

Lisa Eisele [a], Bletë Hulaj [a], Maximilian Podsednik [b],Francesco Laudani [c], Pablo Ayala [d], Alexey Cherevan [d], Annette Foelske [c], Andreas Limbeck [e], Dominik Eder [d*] and Katharina Bica-Schröder [a*]

Contents

S1 Fourier transform infrared spectroscopy of synthesized materials: 2

S2 Thermogravimetric analysis of polymers: 2

S3 XPS Survey spectra of CLP-1: 3

S4 XPS detailed scans of Cl 2p: 3

S5 XPS detailed scans of N 1s: 4

S6 LA-ICP-MS scan lines: 4

S7 EDX scans of sample particles: 5

S8 UV-Vis spectroscopy of aged reaction solution: 5

S9 Calculations of Reaction parameters: 5

S1 Fourier transform infrared spectroscopy of synthesized materials:

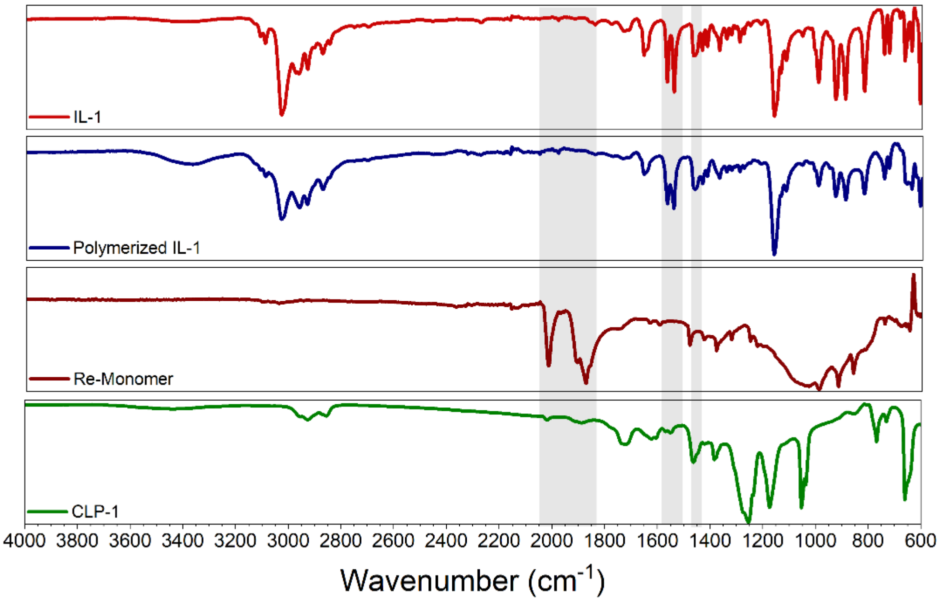

Figure S1: FT-IR spectroscopy of ionic liquid (IL-1)(red), polymerized ionic liquid (PIL) (blue), Re-Monomer (and CLP-1

S2 Thermogravimetric analysis of polymers:

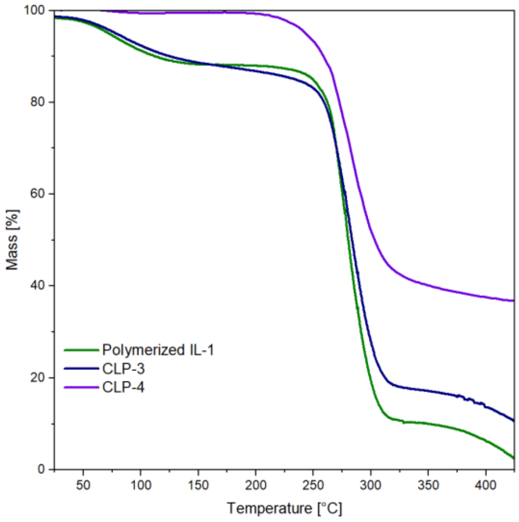

Figure S2: TGA of polymerized IL-1, CLP-3 and CLP-4

S3 XPS Survey spectra of CLP-1:

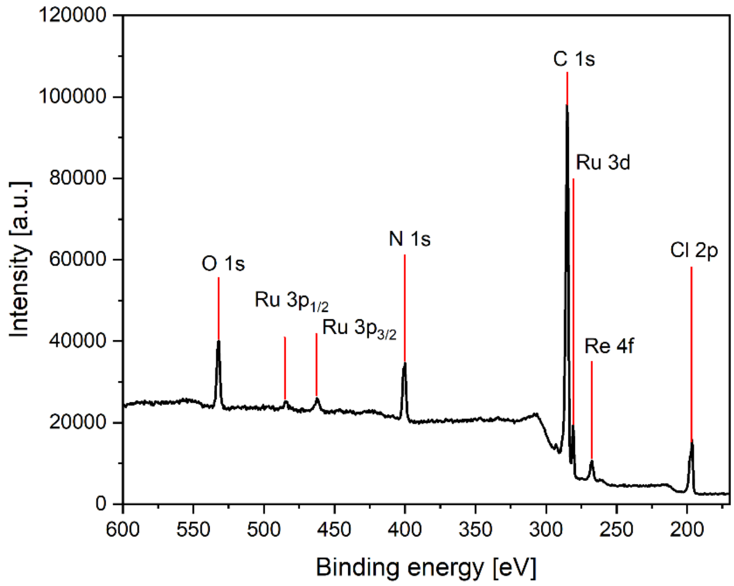

Figure S3: XPS Survey spectra of CLP-1.

S4 XPS detailed scans of Cl 2p:

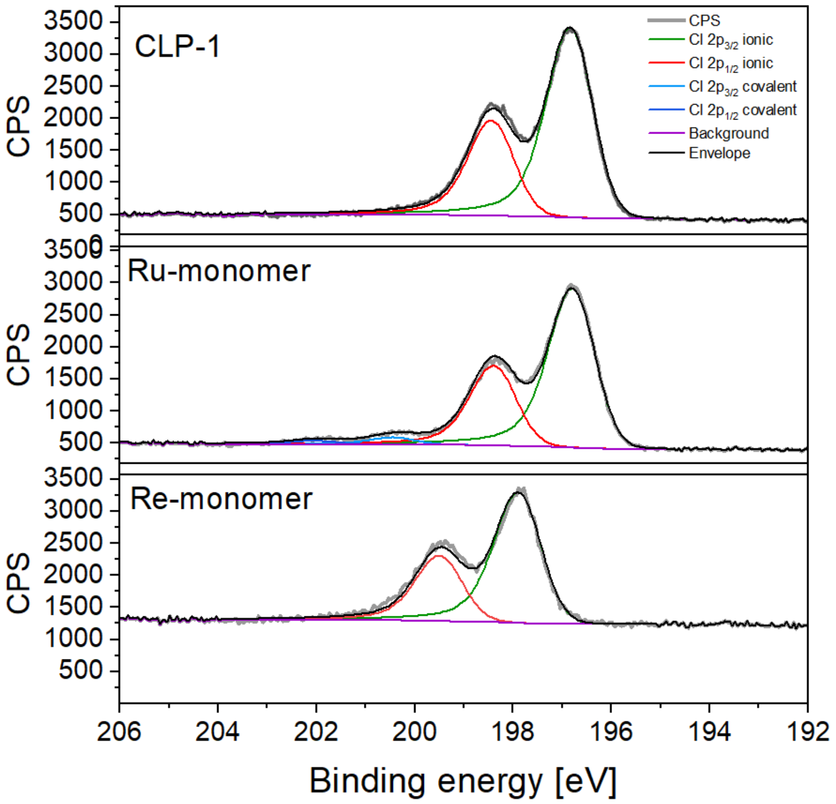

Figure S4: XPS detailed scans of Cl 2p.

S5 XPS detailed scans of N 1s:

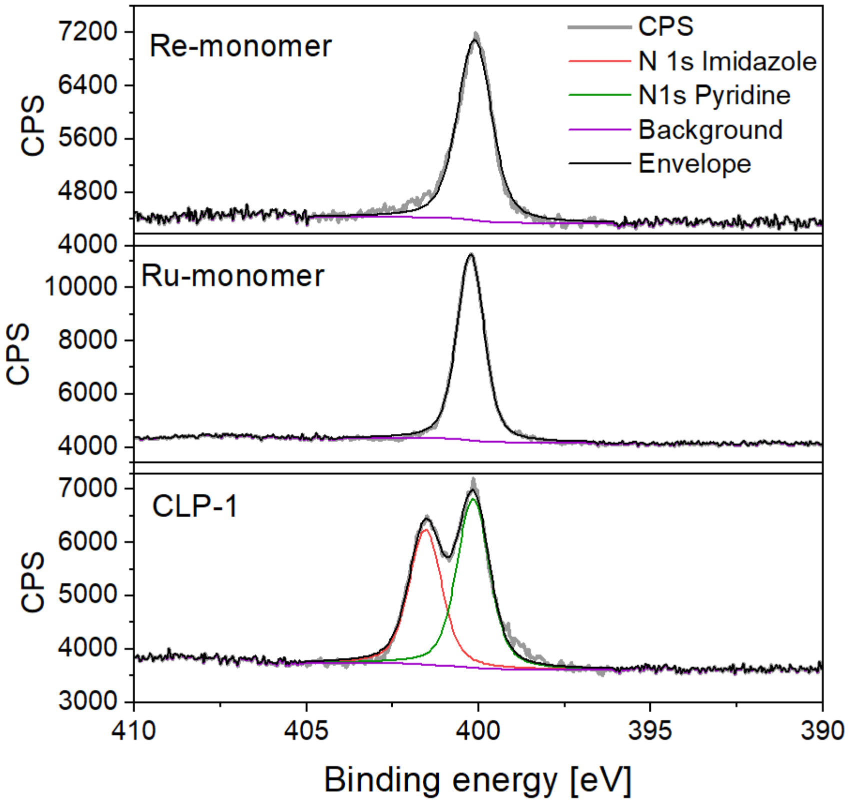

Figure S5: XPS scans of N 1s.

S6 LA-ICP-MS scan lines:

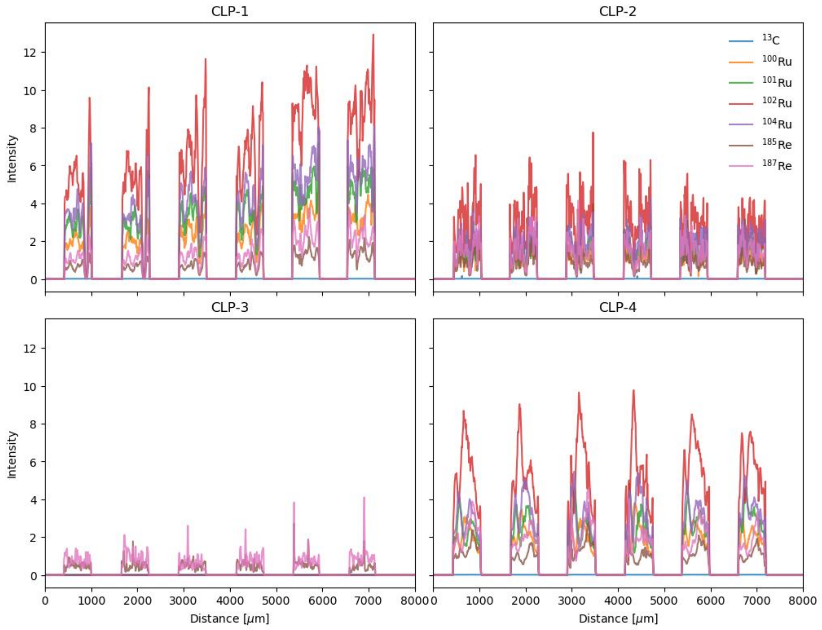

Figure S6: LA-ICP-MS Scans of isotopes in polymers CLP-1 to CLP-4

S7 EDX scans of sample particles:

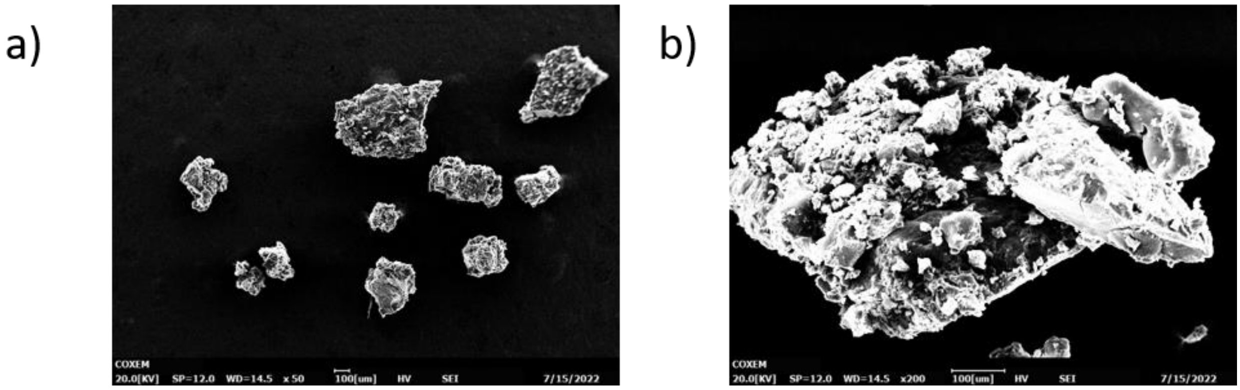

Figure S7: EDX Scans of sample particles of CLP-1. Several particles (a) and closeup (b).

S8 UV-Vis spectroscopy of aged reaction solution:

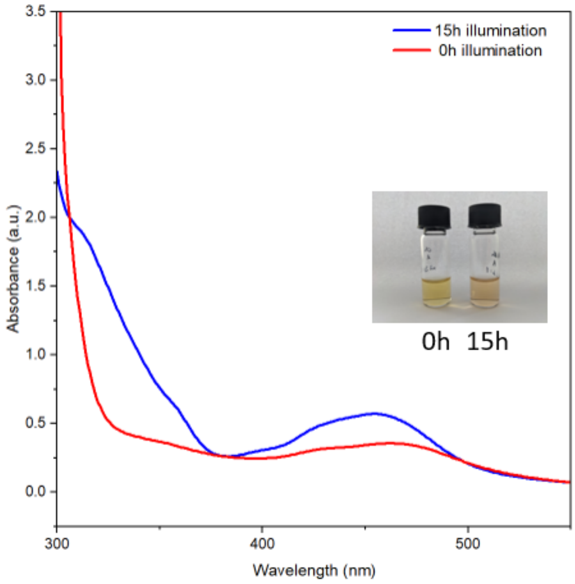

Figure 8: UV Vis spectra of reaction solution after 15h of illumination (blue) and 0h of illumination (red).

S9 Calculations of Reaction parameters:

Reactor parameters:

Reactor volume (ml)	3.7
Reaction volume (ml)	1.5
Reactor headspace (ml)	2.2
Reaction time (sec)	53000

Conversion from ppm to Gas volume in mL:

$$Gas\ volume\ in\ mL = \frac{ppm \times Reactor\ headspace\ (mL)}{10^9}$$

Calculation of gas amount in moles by ideal gas law:

$$n(\mu mol) = \frac{pV}{RT}$$

With parameters:

P is pressure = 101.325 Pa

R is gas constant = 8.314 J mol^{-1} K^{-1}

T is temperature = 295 K

Turnover number (TON) is calculated as following:

$$TON = \frac{nomber\ of\ moles\ of\ CO}{number\ of\ moles\ of\ catalyst}$$

The turnover frequency (TOF) can be calculated from TON divided by the reaction time in minutes:

$$TOF\ (min^{-1}) = \frac{TON}{time\ [min]}$$

The instant TOF is defined as the first derivative (d) of TON against time:

$$Instant\ TOF(min^{-1}) = \frac{d\ (TON)}{d(time\ )}$$
